# Microglia TREM1-mediated neuroinflammation contributes to central sensitization via the NF-κB pathway in a chronic migraine model

**DOI:** 10.1186/s10194-023-01707-w

**Published:** 2024-01-05

**Authors:** Songtang Sun, Zhenzhen Fan, Xuejiao Liu, Longde Wang, Zhaoming Ge

**Affiliations:** 1 Department of Neurology, Lanzhou University Second Hospital, Lanzhou University, Lanzhou, 730000 China; 2 Gansu Provincial Neurology Clinical Medical Research Center, Lanzhou University Second Hospital, Lanzhou, 730000 China; 3 Expert Workstation of Academician Wang Longde, Lanzhou University Second Hospital, Lanzhou, 730000 China

**Keywords:** TREM1, Microglia, Chronic migraine, NF-κB, Central sensitization

## Abstract

**Background:**

Neuroinflammation, mediated by the activation of microglia, contributes to central sensitization, which is associated with the development of chronic migraine (CM). TREM1 receptors amplify the inflammatory response. However, their relationship to CM is unclear. Thus, this study endeavoured to elucidate the exact role of TREM1 in CM.

**Methods:**

Nitroglycerin (NTG) was repeatedly administered intraperitoneally to establish the CM model. Mechanical and thermal sensitivities were assessed using von Frey filaments and hot plate assays. Using Western blotting, TREM1, NF-κB pathway, NLRP3 inflammasome components, and proinflammatory cytokines were all detected. Immunofluorescence was used to examine the cellular distribution of TREM1 and NLRP3, the number of microglia, immunoreactivity, and morphological changes. We examined the effects of TREM1 antagonists (LR12) and NF-κB inhibitors (PDTC) on pain behaviour, as well as the production of c-fos and CGRP. Additionally, we investigated whether LR12 and PDTC affect the activation of microglia and the NLRP3 inflammasome. We synthesized siRNA and TREM1-overexpressing plasmids to transfect BV2 cells treated with LPS and normal BV2 cells and treated TREM1-overexpressing BV2 cells with PDTC. The NF-κB pathway, NLRP3 inflammasome components, and proinflammatory cytokines were quantified using Western blotting.

**Results:**

Following NTG administration, the expression of TREM1 was significantly upregulated and exclusively localized in microglia in the TNC, and was well co-localized with NLRP3. Furthermore, activation of the classical NF-κB pathway was observed. Pre-treatment with LR12 and PDTC effectively attenuated mechanical hypersensitivity, suppressed the expression of c-fos and CGRP, and inhibited NF-κB activity in CM mice. Additionally, inhibition of TREM1 and NF-κB activity mitigated NTG-induced microglia and NLRP3 activation, as well as proinflammatory cytokines production. In vitro, knockdown of TREM1 resulted in attenuated activation of the NF-κB pathway following lipopolysaccharide (LPS) treatment and reduced expression of NLRP3 inflammasome components as well as proinflammatory cytokines. After TREM1 overexpression, the NF-κB pathway was activated, NLRP3 inflammasome components and proinflammatory cytokines were upregulated, and PDTC reversed this phenomenon.

**Conclusions:**

Our findings suggest that TREM1 regulates microglia and NLRP3 activation via the NF-κB pathway, thereby contributing to central sensitization and implicating its involvement in chronic migraine pathogenesis.

**Supplementary Information:**

The online version contains supplementary material available at 10.1186/s10194-023-01707-w.

## Background

Chronic migraine represents a significant neurological disorder that can result in substantial disability [1, 2], imposing a significant burden on both individuals and society [3]. Unfortunately, the treatment outcomes for CM are unsatisfactory, and the use of preventive drugs often leads to significant side effects [4, 5]. Therefore, enhancing our understanding of the precise pathogenesis of CM is crucial to identifying more effective therapeutic targets.

The development of CM is linked to the central sensitization process occurring in the Trigeminal nucleus caudalis [6, 7]. A series of studies have demonstrated that activated microglia release inflammatory mediators and neurotrophic factors, leading to central sensitization of CM, this process involves interactions between microglia and neurons [8–11].

TREM1 (a trigger receptor 1 expressed on myeloid cells) is widely expressed in myeloid cells (monocytes, granulocytes) and microglia [12–14]. TREM1 can aggravate neuroinflammatory injury [15]. Blocking TREM1 can reduce the production of inflammatory factors and ameliorate neuropathic pain [16]. Nevertheless, the involvement of TREM1 in CM remains unexplored.

NF-κB plays a pivotal regulatory role in modulating the activation of microglial and inflammatory response [17, 18]. TREM-1, which binds to the adaptor DNAX activating protein 12 (DAP-12), regulates the activity of NF-κB signalling [19]. The transcription regulation of the NLRP3 inflammasome is associated with NF-κB activity [20, 21]. Inhibition of NLRP3 inflammasome-related pathways has been demonstrated to effectively prevent microglial polarization towards proinflammatory phenotypes [22]. Furthermore, the contribution of NLRP3 activation to CM has been established in studies [23].

Based on these data, we hypothesized that TREM1 activates TNC microglia and NLRP3 inflammasome via NF-κB pathway, participates in central sensitization, and contributes to the occurrence of CM. We validated these findings in vitro and in vivo by using antagonists of these proteins, small interfering RNA, and overexpression plasmids, establishing the unique role of microglial TREM1 in CM mice.

## Methods

### Animals

Male C57BL/6 mice (20-25 g) were procured from Lanzhou University (Lanzhou, China) and kept under controlled conditions with a 12-hour light-dark cycle. They were given unrestricted access to food and water. All experimental procedures strictly adhered to the guidelines established by the National Institutes of Health and received approval from the local animal ethics committee.

### Animal model of chronic migraine

We established a chronic migraine model based on reliable literature [24]; the original concentration of nitroglycerin (NTG) (Beijing Yimin, China) was 5 mg/ml, consisting of 30% ethanol, 30% propylene glycol, and water. Prior to modelling, the original solution was diluted into a working solution of 1 mg/ml and used immediately after preparation. For the NTG group, mice were intraperitoneally administered 10 mg/kg NTG once every other day for a total of five administrations. The control group received only intraperitoneal injections of an equivalent volume of solvent.

### Drug administration

To reveal the effect of TREM1 in chronic migraine (CM), we administered the TREM1 antagonist LR12 (50 mg/kg) (MCE, USA) 30 minutes prior to each NTG or solvent injection, following a once every other day schedule for a total of five administrations. To explore the potential involvement of NF-κB in CM, animals were intraperitoneally injected with PDTC (40 mg/kg) (MCE, USA), an antagonist targeting the NF-κB pathway. Both LR12 and PDTC were diluted using 0.9% normal saline solution, while an equal volume of 0.9% normal saline served as the control solution. The dosage selection of LR12 and PDTC presented in this paper is based on the findings from our preliminary experimental study, with an additional file providing more comprehensive data (Additional file 1: Fig. S1).

### Cell culture and treatment

BV2 cells were obtained from Wuhan Procell Life Science & Technology Co., Ltd. and cultured in medium containing 10% foetal bovine serum and 5% penicillin‒streptomycin. The BV2 cell model was established by exposing the cells to LPS (MCE, USA), to determine the optimal concentration of LPS, cells were treated with LPS at 0,1,5,10 μg/ml, while suppressing TREM1 expression through transfection of small interfering RNA (siRNA). Additionally, TREM1 plasmids were introduced into BV2 cells to enhance its expression. To inhibit the NF-κB pathway, PDTC (100 μM) treatment was administered for 24 hours after overexpressing TREM1 in the cells. Each experiment was independently repeated at least six times.

### Cell transfection

For the TREM1 knockdown experiment, three siRNA sequences were designed: their oligonucleotides were as follows: siRNA-1 (GCUGCCAUUGUUCUAGAGGAATT), siRNA-2 (CUGUCAUCAUUCCUAUUACAATT), and siRNA-3 (CCACAUCCAGUGUUACUAUUUTT). A negative control siRNA was also employed as the experimental control. The knockdown efficiency was assessed through Western blot, and the siRNA with the highest knockdown efficiency was chosen for further experiments. To induce TREM1 overexpression, pcDNA3.1-TREM1 overexpression vector (oe-TREM1) and pcDNA3.1-NC vector (oe-NC) were synthesized. Transfection efficiency was evaluated using Western blot. The interfering RNA and TREM1 plasmid were obtained from Sangon Biotech (Shanghai, China), while Lipofectamine® 3000 reagent (Invitrogen) was used as the transfection reagent.

### Behavioural assessment

We conducted behavioural tests under low-light conditions. Before the formal behavioural experiment, the mice underwent a 3-day period of behavioural training. The researchers who conducted the behavioural tests were blinded to the experimental grouping. Although NTG is a vasodilator, no significant hypotensive effects were found during the experiment.

Migraine is often accompanied by cephalalgia, while aberrant cutaneous discomfort indicates central sensitization. To emulate the features of migraine, we measured mechanical hind paw and periocular pain, as well as latency to withdraw from a thermal stimulus. Prior to experimentation, mice were allowed to acclimate for 30 minutes. The thresholds for mechanical and thermal pain were measured before and 2 hours after each administration, respectively. For the mechanical threshold test, mice were placed in a specially designed glass box, and their hind paws on the same side were vertically stimulated with corresponding von Frey filaments (ranging from 0.008 g to 2 g), avoiding contact with the footpad. Indicators such as paw withdrawal, trembling, or grooming behaviour were noted to ascertain the presence of a positive reaction. To evaluate their mechanical sensitivity in the periorbital area, mice were gently placed within a confined enclosure, and Von Frey monofilaments were meticulously applied in an upright manner. Immediate head retraction upon stimulation or facial scratching with the front paw on the same side was considered indicative of a favourable response during this specific test. Finally, we utilized an online resource available at https://bioapps.shind-apps.io/von-Frey-app/ to calculate and determine their 50% mechanical threshold.

Thermal sensitivity was measured using a hot plate experiment. The mice were placed on the hot plate instrument in advance, and when they adapted to the environment, the switch was turned on. If the mice lifted their paws, licked their feet, or shook themselves, it was regarded as a positive reaction. The time was recorded and measured three times, with an interval of at least 10 minutes between each measurement.

### Western blot analysis

TNC tissue was collected 6 hours after drug injection, while for c-fos, it was collected 2 hours after drug injection. We fully anaesthetized the mice with 5% pentobarbital and then quickly extracted the TNC and stored it at -80°C for preservation. After fully homogenizing the tissue in RIPA lysis buffer, we centrifuged it at 14000 rpm for 15 minutes. Following quantification of protein concentration using the BCA kit (Beyotime, China), we loaded and separated 40 μg of protein on an SDS-polyacrylamide gel and transferred them onto NC membranes. To block any nonspecific binding, we incubated the membranes with 5% skimmed milk for one hour at room temperature before incubating overnight at 4°C with the corresponding primary antibodies in (Table 1). Following membrane rinsing, the membranes were incubated with the corresponding species of secondary antibodies conjugated with horseradish peroxidase in (Table 1). Detection was performed using a gel imaging system (Bio-Rad, USA), with β-actin serving as a reference.

**Table 1:** Antibody used in western blotting and immunofluorescence staining

**Antibody**	**Manufacturer**	**Catalog number**	**Host**	**Dilution**
**For western blot**				
TREM1	Abcam, UK	ab217161	Rabbit	1:1000
c-fos	Santa Cruz, USA	sc166940	Mouse	1:500
CGRP	Abcam, UK	ab139264	Rabbit	1:1000
p-p65	CST, USA	3033	Rabbit	1:1000
p-65	CST, USA	8242	Rabbit	1:1000
p-IκBα	CST, USA	2859	Rabbit	1:1000
IκBα	CST, USA	4814	Mouse	1:1000
NLRP3	CST, USA	15101	Rabbit	1:1000
ASC	CST, USA	67824	Rabbit	1:1000
Cleaved-Caspase-1	Proteintech, China	22915-1-AP	Rabbit	1:2000
Mature IL-1β	Abcam, UK	ab234437	Rabbit	1:1000
Mature IL-18	Abcam, UK	ab191860	Rabbit	1:2000
β-actin	Santa Cruz, USA	sc-47778	Mouse	1:1000
HRP conjugated anti-rabbit	Proteintech, China	SA00001-2	Goat	1:100000
HRP conjugated anti-mouse	Proteintech, China	SA00001-1	Goat	1:100000
**For immunofluorescence**				
TREM1	Abcam, UK	ab217161	Rabbit	1:200
NLRP3	Proteintech, China	68102-1-Ig	Mouse	1:100
CGRP	Santa Cruz, USA	sc-57053	Mouse	1:100
Iba1	Abcam, UK	ab5076	Goat	1:500
GFAP	Santa Cruz, USA	sc-33673	Mouse	1;200
NeuN	Abcam, UK	ab104224	Mouse	1:1000
Alexa Fluor 488 Donkey anti-mouse IgG	Jackson ImmunoResearch, USA	715-545-150	Donkey	1:200
Alexa Fluor 594 Donkey anti-mouse IgG	Jackson ImmunoResearch, USA	715-585-150	Donkey	1:200
Alexa Fluor 488 Donkey anti-rabbit IgG	Jackson ImmunoResearch, USA	711-545-152	Donkey	1:500
Alexa Fluor 594 Donkey anti-goat IgG	Jackson ImmunoResearch, USA	705-585-147	Donkey	1:500

### Immunofluorescence staining

The mice were deeply anaesthetized prior to cardiac perfusion with precooled 0.9% normal saline and 4% paraformaldehyde (PFA), followed by brain extraction and subsequent fixation in a 4% PFA solution. The medullary segment containing the TNC was taken and sequentially immersed in 20% and 30% sucrose/PBS solution for gradient dehydration. After being frozen, the tissue was sliced into sections that were only 10 μm thick using a low-temperature cryostat (Leica, Japan). To prepare for further analysis, the slices were first blocked with 5% BSA before undergoing overnight incubation with the corresponding primary antibodies in (Table 1). After a thorough wash, the slices were subjected to incubation with fluorescent secondary antibodies in (Table 1) at room temperature for 1 hour. Subsequently, DAPI staining was conducted for 10 minutes. Images were obtained using a confocal microscope (Nikon AXR, Japan). The TNC region was determined based on the mouse brain atlas, and we employed ImageJ software (version 2.3.0, USA) for immunofluorescence quantification analysis. CGRP immunoreactivity was assessed by calculating the percentage of CGRP-positive area. To quantify the expression levels of Iba1-positive microglia, a square image measuring 295×295 μm^2 was obtained from the superficial layer of the TNC, and all immune response cells in this region were quantified. When analyzing the morphological characteristics of microglia, we utilized Neuron J, a plug-in capable of tracking microglial process trajectories, to measure the total and mean initial process length of microglia. For each mouse, three slices were taken, and within each slice, 4-6 fields of view were captured (*n*=5). We performed analysis on both the left and right sides of each slice, and subsequently calculated the average value of both sides for further statistical analysis.

### Statistical analysis

The statistical analysis was conducted using Prism 9.5.1 software (GraphPad Software Inc., San Diego, CA, USA). The data are presented as the mean±SEM. Pairwise comparisons were employed using one-way ANOVA followed by Tukey's post hoc test, while two-way ANOVA with Tukey's post hoc tests was utilized for analysing data from multiple groups. Statistical significance was defined as *p*<0.05.

## Results

### TREM1 is expressed in TNC microglia and is highly upregulated after repeated NTG administration

**Fig. 1:**
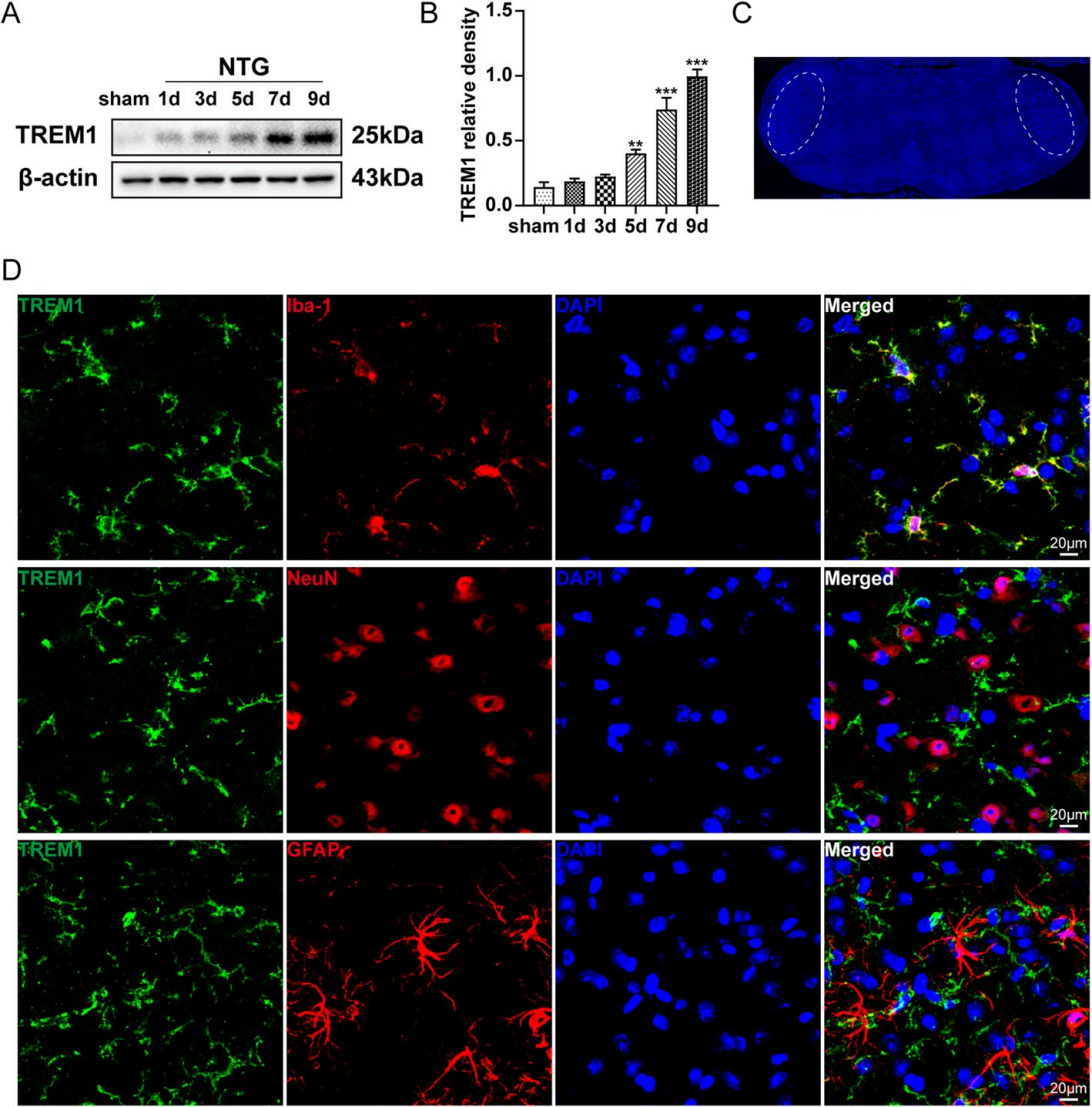
TREM1 expression is highly upregulated in TNC microglia following repeated NTG administration. **a, b** Immunoblot analysis of TREM1 at the indicated times following NTG administration (*n*=6). **c** The white dashed box represents the TNC area. **d** Costaining of TREM1 with Iba1, NeuN, and GFAP within the TNC region. Scale bar: 20 μm. Values are the mean±SEM; one-way ANOVA and Tukey's post hoc tests; **p < 0.01 and ***p < 0.001 vs. the sham group

To elucidate the effect of TREM1 in CM, we investigated the expression pattern and temporal progression of TREM1 after repeated NTG administration. Immunoblot analysis revealed a gradual increase in TREM1 expression over time, with a significant difference observed on day 5 and reaching its peak on day 9 (Fig. 1a, b). Through double immunostaining using Iba1, NeuN, and GFAP markers, we found exclusive expression of TREM1 in microglial cells without detection in neurons or astrocytes (Fig. 1d). Therefore, we conclude that TREM1 is present in TNC microglia and upregulated in CM mice.

### Blocking TREM1 alleviates CM-related pain hypersensitivity in CM mice

**Fig. 2:**
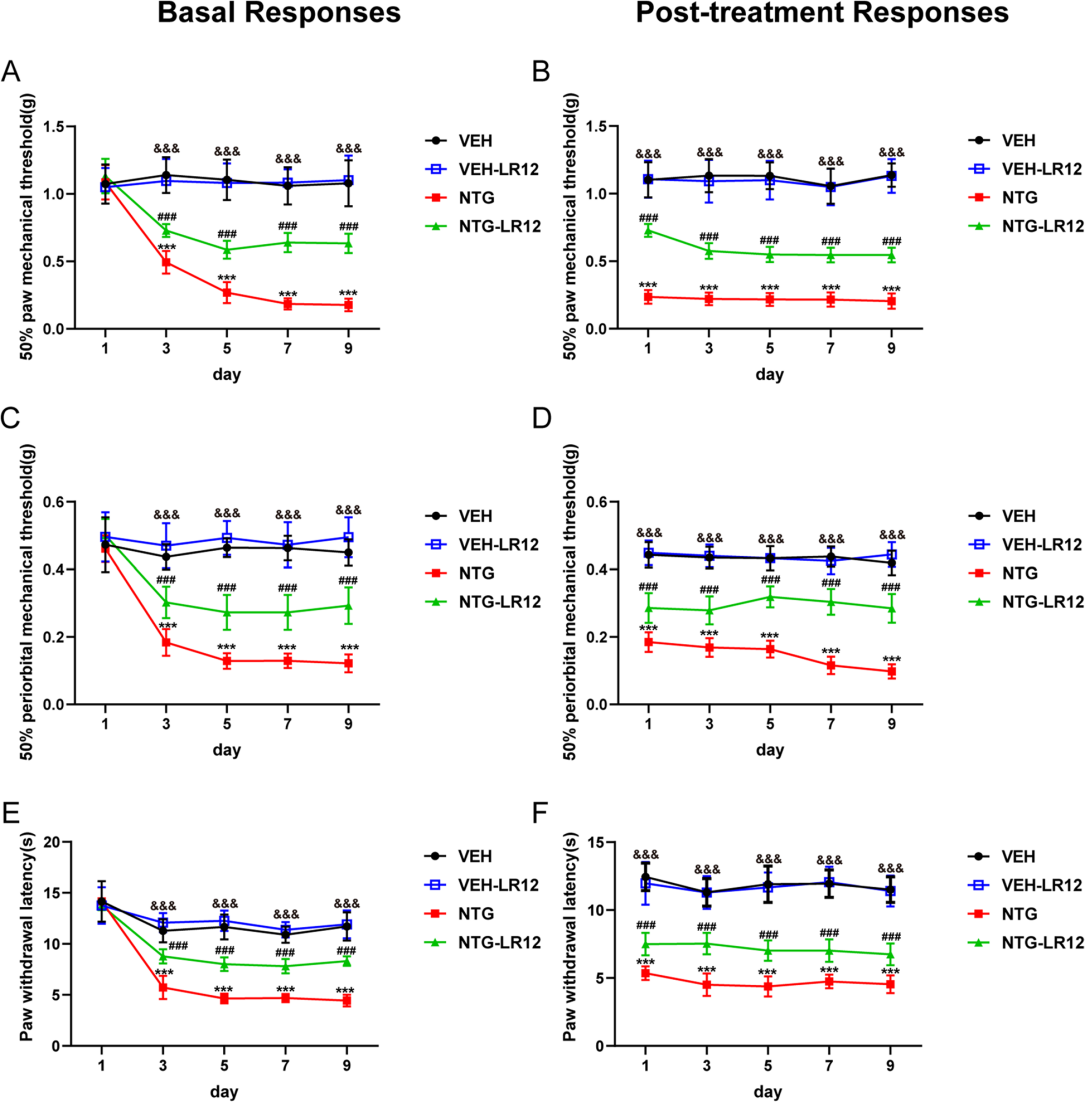
Blocking TREM1 alleviates pain hypersensitivity in CM mice. LR12 effectively prevented the decrease in both basal and acute mechanical withdrawal thresholds of the hind paw (a, b) and periorbital area (c, d) and extended the heat withdrawal latency (e, f) (*n*= 10). Acute hypersensitivity reactions were detected 2 hours after each NTG treatment. Values are the mean±SEM; two-way ANOVA and Tukey's post hoc tests; ***p < 0.001 vs. the VEH group; ^###^*p* < 0.001 vs. the NTG group; ^&&&^*p* < 0.001, the NTG-LR12 group vs. the VEH-LR12 group

Clinically, migraine patients frequently exhibit aberrant cutaneous pain sensations [25], a phenomenon also observed in a chronic migraine model induced by NTG [26]. To explore the potential involvement of TREM1 in TNC microglia in CM-related pain, we injected the TREM1-specific antagonist LR12 (50 mg/kg) intraperitoneally every other day before NTG administration. Behavioural data showed that LR12 significantly increased the basal and acute mechanical withdrawal thresholds and prolonged the heat withdrawal latency compared to the NTG group but had no effect on the VEH group mice (Fig. 2). Our data demonstrated that the inhibition of TREM1 could exhibit a partial efficacy in mitigating pain hypersensitivity in migraine mice, while exhibiting no discernible impact on normal mice.

### TREM1 blockade results in downregulation of c-fos and CGRP expression

**Fig. 3:**
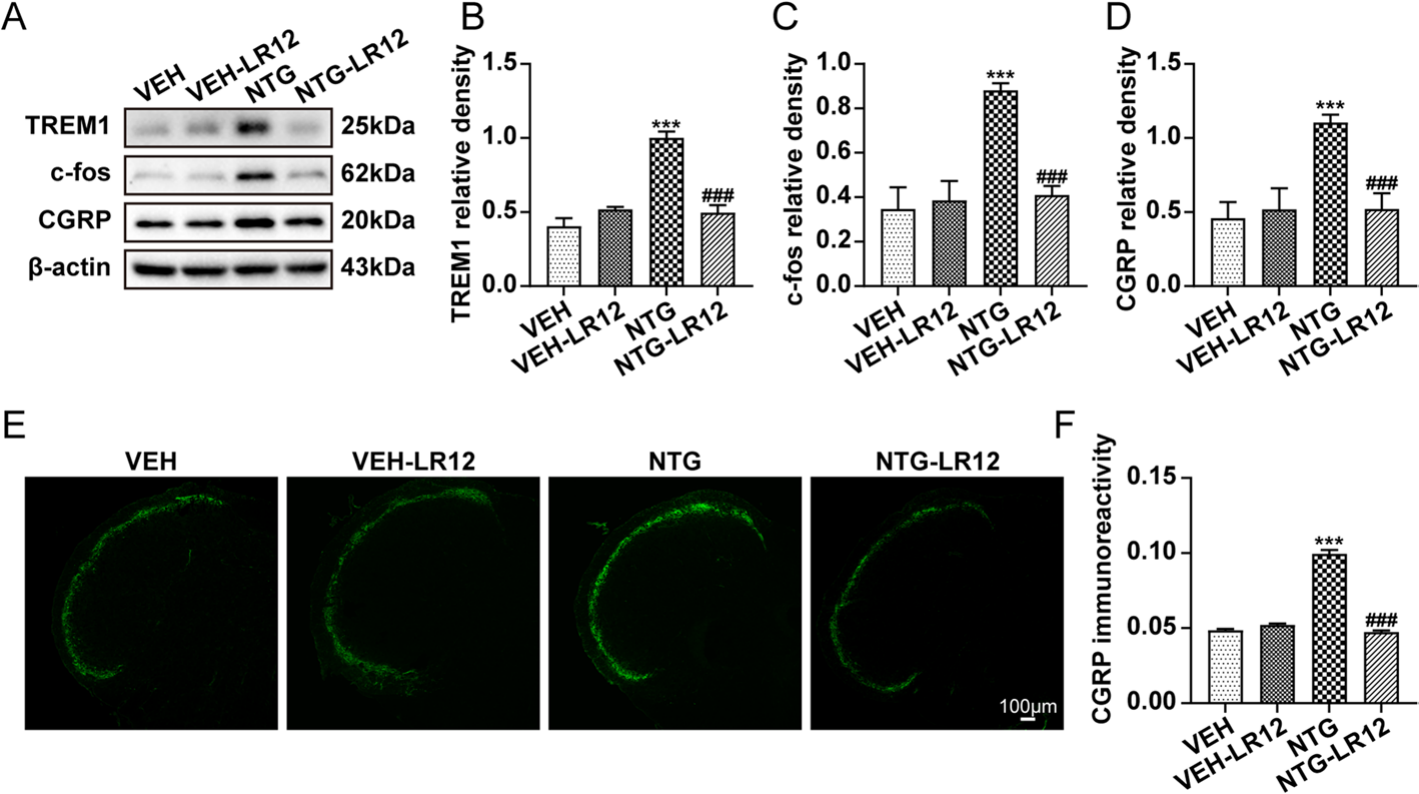
TREM1 blockade results in downregulation of c-fos and CGRP expression. **a-d** Immunoblot analysis of TREM1, c-fos, and CGRP (*n*=6). **e**, **f** Quantification of CGRP immunoreactivity (*n*=5). Scale bars: 100 μm; each mouse was selected for three slices for analysis. Values are presented as the mean ± SEM; one-way ANOVA and Tukey's post hoc tests; ***p < 0.001 vs. the VEH group; ^###^*p* < 0.001 vs. the NTG group

To investigate the potential involvement of TREM1 in central sensitization, LR12, a specific antagonist of TREM1, was administered to mice. Immunoblot analysis revealed that LR12 significantly downregulated the expression of TREM1, c-fos, and CGRP (Fig. 3a-d). Quantification of CGRP immunofluorescence staining revealed a significant reduction in CGRP immunoreactivity upon treatment with LR12 (Fig. 3e, f). These findings provide compelling evidence for the involvement of TREM1 in central sensitization and its contribution to the development of CM.

### TREM1 modulates the activation of TNC microglia and inflammatory response after NTG administration

**Fig. 4:**
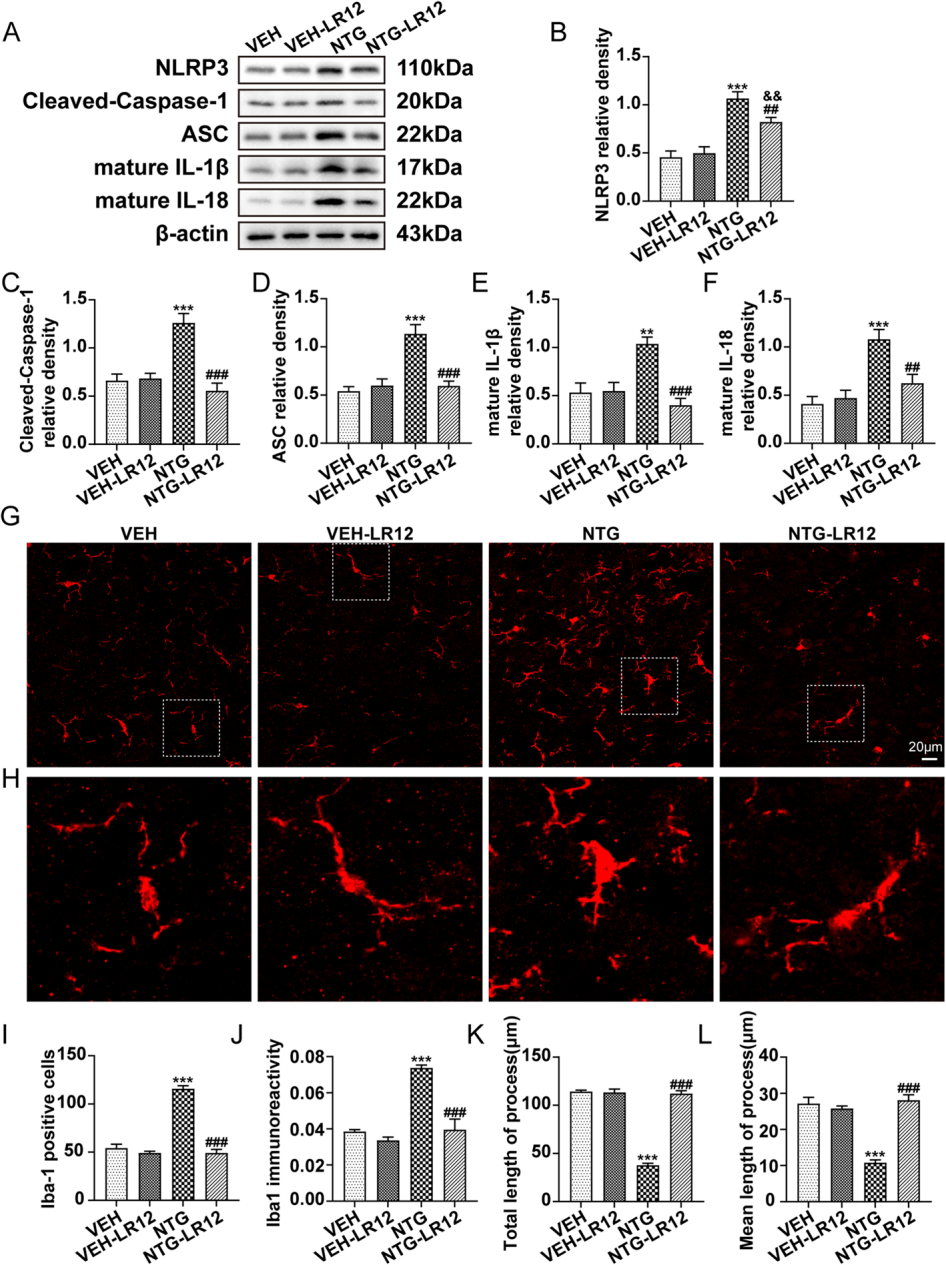
TREM1 is critical for the activation of TNC microglia and inflammatory response after NTG administration. **a-f** Immunoblot analysis was conducted to detect the expression levels of NLRP3, cleaved-caspase-1, ASC, mature IL-1β, and mature IL-18 after the administration of NTG and LR12 (*n* = 6). **g** Immunofluorescence images of Iba1 in the TNC of mice treated with NTG and LR12. **h** The magnified image of Iba-1 corresponds to that within the white dotted line frame. **i–l** Immunofluorescence quantification was performed to measure Iba-1-positive microglia, Iba1 immunoreactivity, total neurite length per microglia, and mean initial neurite length per microglia (*n* = 5). Three slices were taken from each mouse for analysis, with each slice containing 4-6 FOVs. Scale bar: 20 μm. The values are the mean ± SEM; one-way ANOVA and Tukey's post hoc tests; **p < 0.01 and ***p < 0.001 vs. the VEH group; ^##^*p* < 0.01, ^###^*p* < 0.001 vs. the NTG group; ^&&^*p* < 0.01 vs. the VEH-LR12 group

The involvement of microglial activation is widely acknowledged for its substantial contribution to the regulation of the inflammatory response, which is pivotal in pain-related disorders and the pathogenesis of migraine [27, 28]. To elucidate the regulatory function of TREM1 in microglial activation and inflammatory response, we utilized immunoblots to assess the expression of NLRP3 inflammasome components and immunofluorescence techniques to assess the number and morphological changes of microglia as well as the expression pattern of NLRP3. Our findings demonstrated that NTG induced the upregulation of NLRP3 inflammasome components, and mature IL-1β and mature IL-18 were remarkably rescued upon inhibition of TREM1 (Fig. 4a-f). Double immunofluorescence staining showed that NLRP3 was mainly present in microglia within the TNC of migraine mice, which is consistent with the findings of Jiying Zhou et al [23], and we found that TREM1 and NLRP3 were well co-localized, indicating that TREM1 may regulate NLRP3 expression through intracellular signalling in TNC microglia (Additional file 2: Fig. S2). Furthermore, quantification of immunofluorescence staining revealed that LR12 effectively decreased the number of microglia and immune reactivity compared to the NTG group (Fig. 4g, i, j). Additionally, NTG administration significantly diminished the total and mean initial process length of microglia, which were reversed by LR12 (Fig. 4h, k, l). Notably, following NTG administration, distinct alterations in microglial morphology were observed, including enlarged cell bodies and shortened processes. However, these morphological changes were effectively attenuated by LR12 administration (Fig. 4h). Collectively, our findings highlight the crucial involvement of TREM1 in mediating microglial activation and inflammatory response after NTG administration.

### TREM1 is required for NF-κB activation in CM mice

**Fig. 5:**
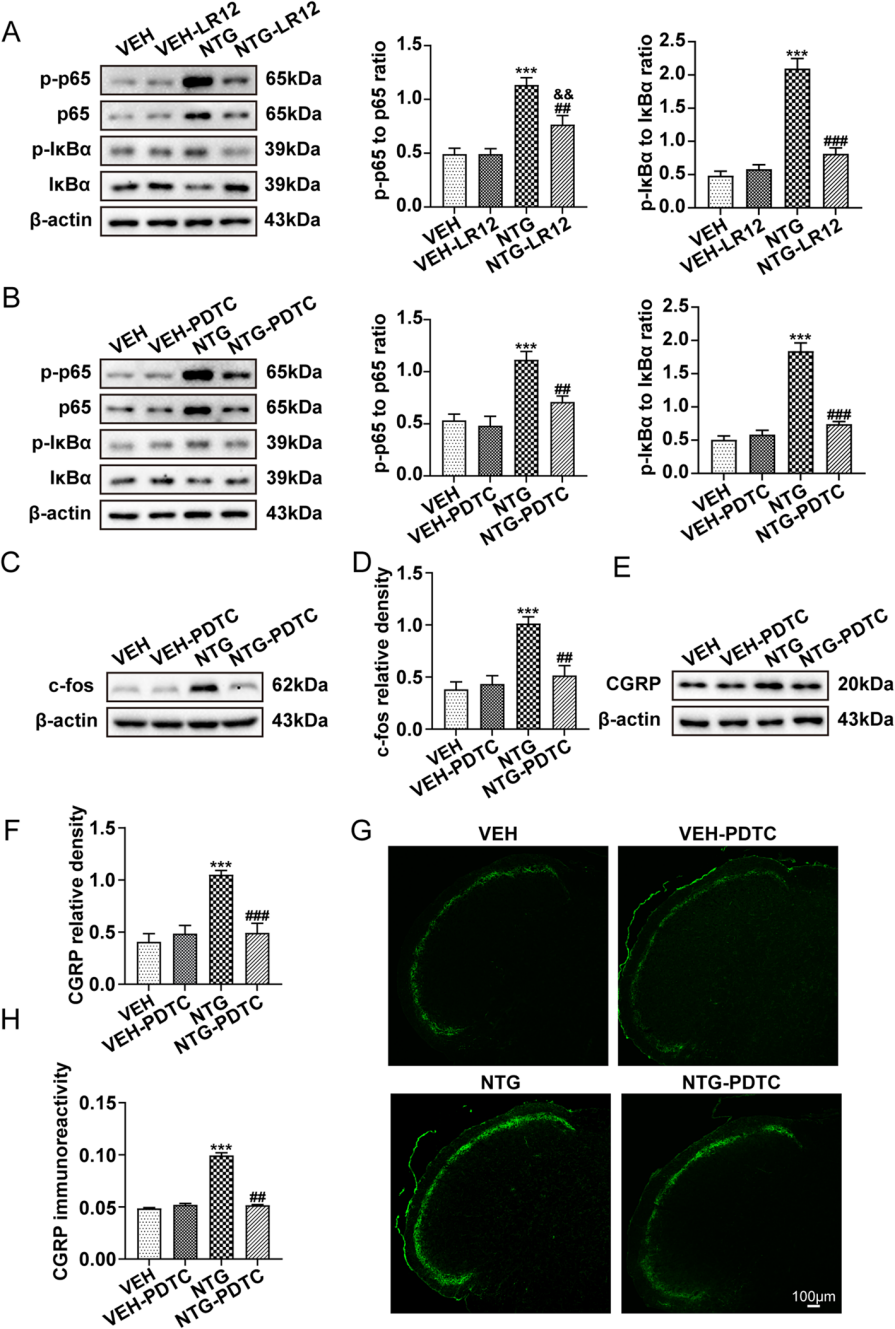
NF-κB inhibition results in downregulation of c-fos and CGRP expression. **a**, **b** Immunoblot analysis of p-p65 to p65 ratio and p-IκBα to IκBα ratio was conducted in the indicated groups (*n*=6). **c-f** Immunoblot analysis of c-fos and CGRP was conducted (*n*=6). **g**, **h** Quantification of CGRP immunoreactivity was conducted (*n*=5). Three slices were taken from each mouse for analysis. Values are the mean±SEM; one-way ANOVA and Tukey's post hoc tests; ***p < 0.001 vs. the VEH group; ^##^*p* < 0.01 and ^###^*p* < 0.001 vs. the NTG group; ^&&^*p* < 0.01 vs. the VEH-LR12 group.

Studies have substantiated the involvement of TREM1 in NF-κB activation, thereby contributing significantly to neuroinflammation [29]. To elucidate the specific mechanism responsible for the impact of TREM1 in CM mice, LR12, a specific antagonist of TREM1, was administered. Immunoblot analysis showed an upregulation in the ratio of p-p65 to total p65 and p-IκBα to total IκBα, indicating activation of NF-κB following repeated NTG administration, which was reversed by inhibition of TREM1 (Fig. 5a). These findings suggest that TREM1 exerts regulatory control over the modulation of NF-κB activation in CM mice.

### NF-κB inhibition results in downregulation of c-fos and CGRP expression

We have demonstrated that TREM1 regulates NF-κB activity in CM mice. To verify the effect of NF-κB in CM mice, we administered the NF-κB-specific antagonist PDTC (40 mg/kg) prior to each NTG administration. Immunoblot analysis revealed that PDTC significantly suppressed NF-κB signalling activation (Fig. 5b), resulting in a reduction in c-fos and CGRP expression compared to the NTG group (Fig. 5c-f). Additionally, immunofluorescence analysis demonstrated that PDTC effectively attenuated CGRP immunoreactivity compared to the NTG group (Fig. 5g, h). Based on these findings, we conclude that NF-κB mediates central sensitization in CM mice and contributes to its development.

### NF-κB inhibition attenuates CM-related pain hypersensitivity

**Fig. 6:**
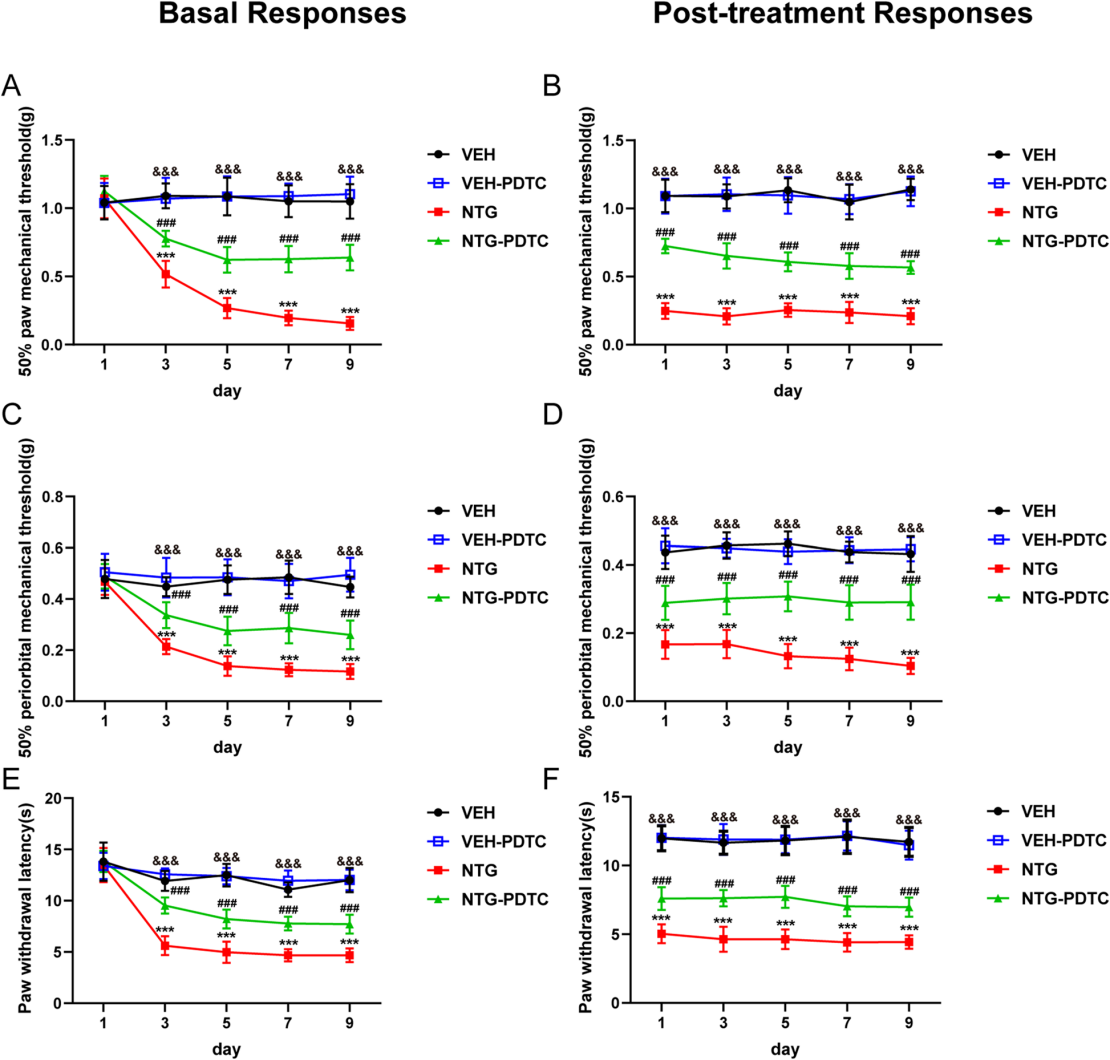
NF-κB inhibition attenuates NTG-induced pain hypersensitivity. PDTC effectively prevented the decrease in both basal and acute mechanical withdrawal thresholds of the hind paw (a, b) and periorbital area (c, d) and extended the heat withdrawal latency (e, f) . Acute hypersensitivity reactions were detected 2 hours after each NTG treatment. Values are the mean ± SEM; *n* = 10; two-way ANOVA and Tukey's post hoc tests; ***p < 0.001 vs. the VEH group; ^###^*p* < 0.001 vs. the NTG group; ^&&&^*p* < 0.001, the NTG-PDTC group vs. the VEH-PDTC group.

To further validate the impact of the NF-κB pathway on CM, we administered PDTC, a potent antagonist of NF-κB, prior to each NTG injection and assessed its effect on pain hypersensitivity in CM mice. Behavioural data showed that PDTC significantly increased the basal and acute mechanical withdrawal thresholds and prolonged the heat withdrawal latency compared to the NTG group but had no effect on the VEH group mice (Fig. 6). Our study demonstrated that the inhibition of NF-κB activity may effectively alleviated pain hypersensitivity in CM mice to a certain extent, while exhibiting no discernible impact on normal mice.

### NF-κB activity modulates microglial activation and inflammatory response after NTG administration

**Fig. 7:**
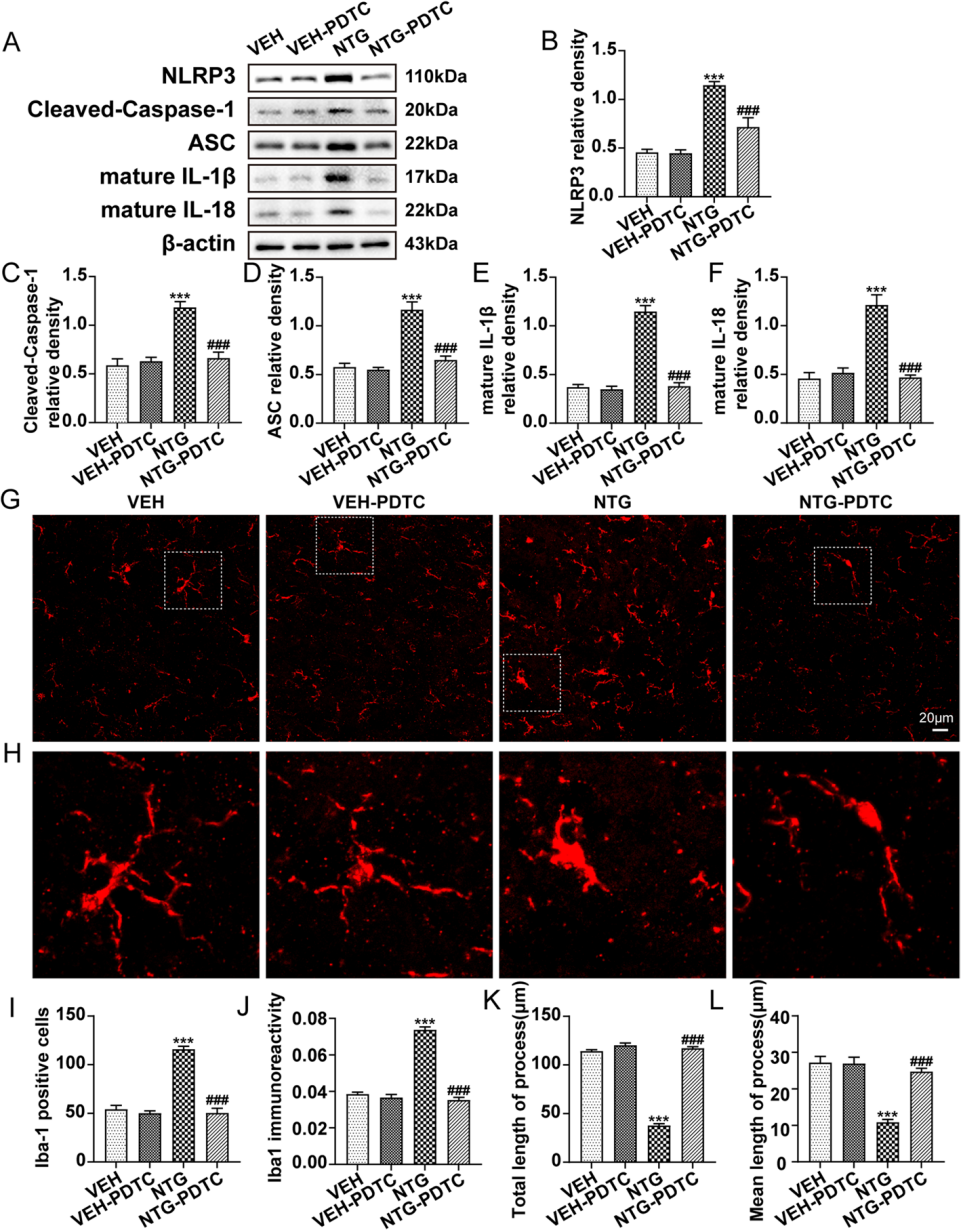
NF-κB activity modulates microglial activation and inflammatory response after NTG administration. **a-f** Immunoblot analysis of NLRP3, cleaved-caspase-1, ASC, mature IL-1β, and mature IL-18 after administration of NTG and PDTC (*n* = 6). **g** Immunofluorescence images of Iba1 in the TNC of mice treated with NTG and PDTC. Scale bar: 20 μm. **h** The magnified image of Iba-1 corresponds to that within the white dotted line frame. **i–l** Immunofluorescence quantification of Iba-1-positive microglia, immunoreactivity, and total and mean initial neurite length per microglia (*n* = 5). Three slices were taken from each mouse for analysis, with each slice containing 4-6 FOVs. Values are the mean ± SEM; one-way ANOVA and Tukey's post hoc tests; ***p < 0.001 vs. the VEH group and ^###^*p* < 0.001 vs. the NTG group

Considering the close association between TREM1 and NF-κB in CM mice, we subsequently investigated whether NF-κB is also essential, similar to TREM1, for microglial activation and the subsequent inflammatory response after NTG administration. We administered PDTC, an NF-κB inhibitor, to mice as previously described. Immunoblot analysis revealed that PDTC pre-treatment significantly attenuated the NTG-induced upregulation of the NLRP3 inflammasome components, mature IL-1β and mature IL-18 (Fig. 7a-f). Furthermore, our immunofluorescence analysis of Iba1 demonstrated that PDTC markedly reduced the number and immunoreactivity of Iba1-positive microglia (Fig. 7g, i, j) while also reversing morphological changes such as cytoplasmic hypertrophy and process shortening compared to the NTG group (Fig. 7h, k, l). These results indicate that NF-κB activity contributes to the activation of microglia and the NLRP3 inflammasome, thereby initiating a subsequent inflammatory response in CM mice.

### TREM1 is essential for the augmentation of NF-κB activity and induction of the NLRP3 inflammasome in BV2 microglia upon LPS stimulation

**Fig. 8:**
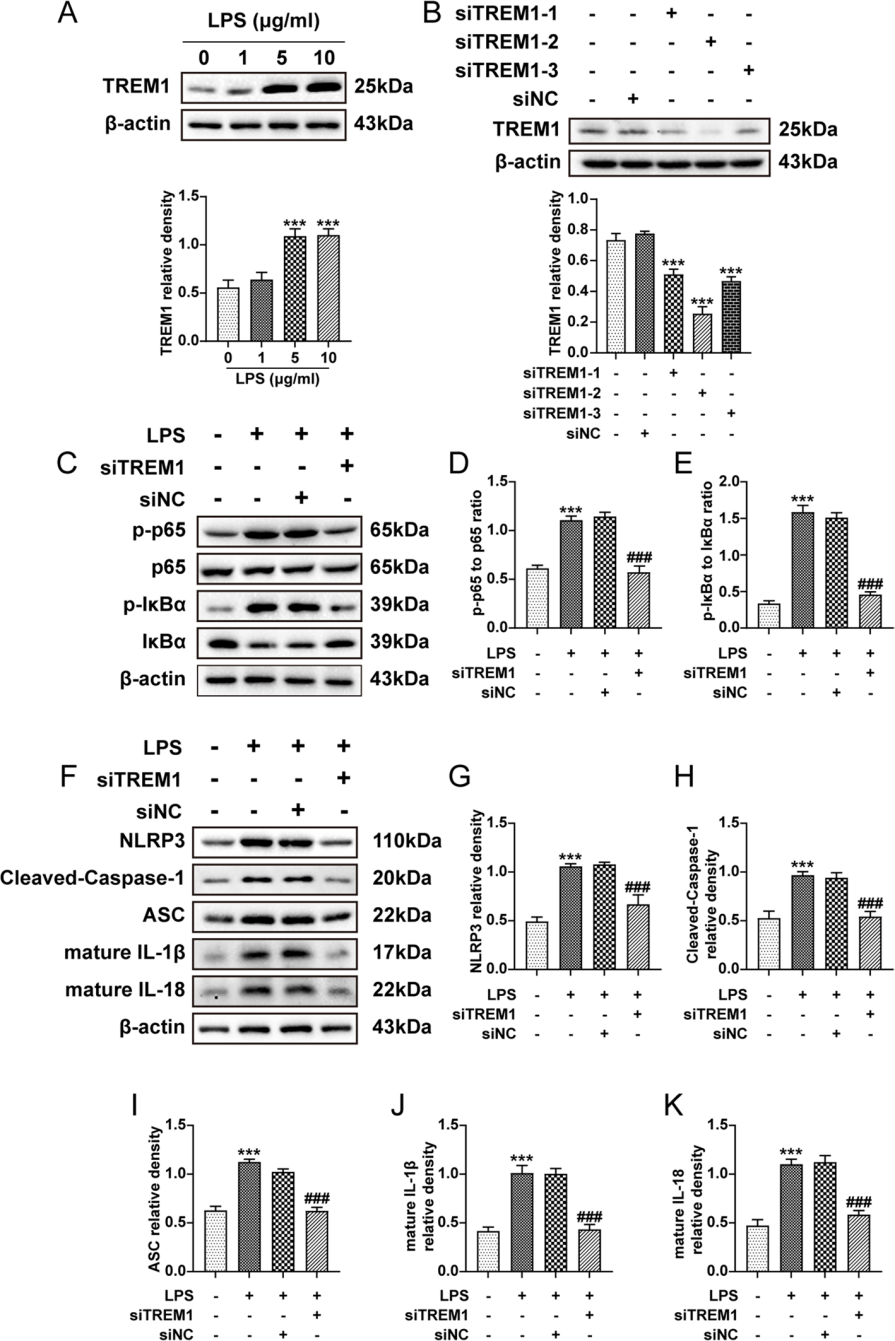
TREM1 modulates NF-κB activity and facilitates the activation of the NLRP3 inflammasome. Following transfection with negative control siRNA (siNC) or TREM1-siRNA (siTREM1) for a duration of 24 hours, the cell culture medium was replaced and subsequently stimulated with LPS (5 μg/ml) for a period of 24 hours prior to harvesting. Immunoblot analysis was performed on BV2 cells in the indicated groups for TREM1 (a, b); Immunoblot analysis was performed on BV2 cells in the sham, LPS, LPS-siNC, and LPS-siTREM1 groups for p-p65 to p65 ratio and p-IκBα to IκBα ratio (c-e) and NLRP3, cleaved-caspase-1, ASC, mature IL-1β, and mature IL-18 (f-k). Values are the mean ± SEM; one-way ANOVA and Tukey's post hoc tests; ***p < 0.001 vs. the control group and ^###^*p* < 0.001 vs. the LPS group

Considering the constraints in systemic administration layout, it is imperative to ascertain the role of TREM1 in microglia through further advancements. After LPS treatment, we found that the expression of TREM1 gradually increased with the concentration of LPS, and reached its peak at 5 μg/ml, while no significant difference between BV2 cells treated with 5 μg/ml and 10 μg/ml (Fig. 8a). We employed small interfering RNA to downregulate the expression of TREM1 in BV2 cells. Immunoblot analysis revealed the expression of TREM1 was effectively downregulated by interfering RNA (Fig. 8b) and treatment with LPS significantly enhanced the p-p65 to p65 and p-IκBα to IκBα ratios, which were subsequently reversed upon knockdown of TREM1 (Fig. 8c-e). Moreover, knockdown of TREM1 attenuated the upregulation of NLRP3 inflammasome components and mature IL-1β and mature IL-18 observed in the LPS group (Fig. 8f-k). Thus, our findings showed that the involvement of TREM1 is pivotal in modulating NF-κB activity and NLRP3 inflammasome activation in BV2 cells stimulated with LPS.

### TREM1 modulates the activation of the NLRP3 inflammasome via the NF-κB pathway in BV2 cells

**Fig. 9:**
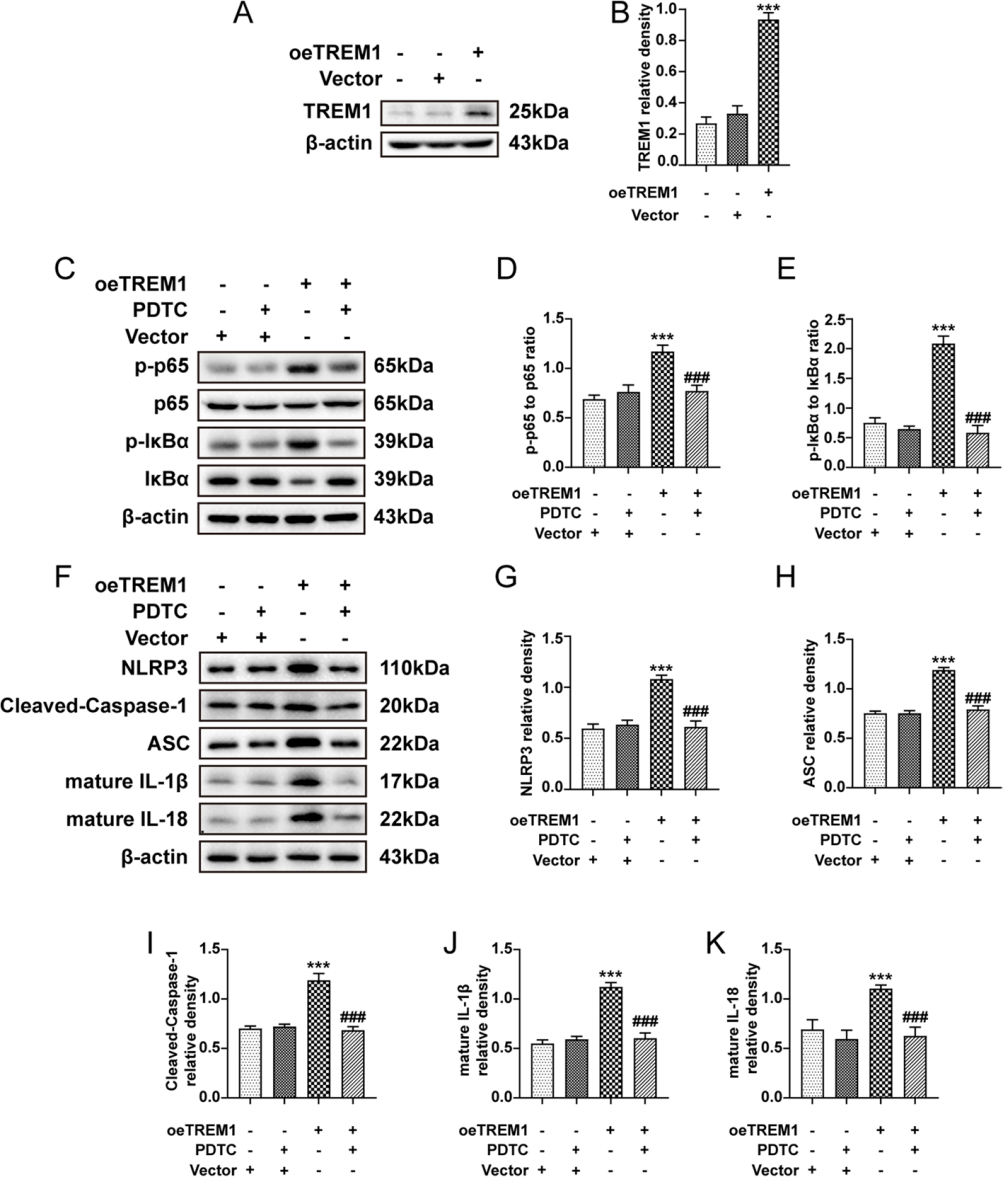
TREM1 modulates the activation of the NLRP3 inflammasome via the NF-κB pathway in BV2 cells. After transfection with either overexpression negative control vector or overexpression TREM1 (oeTREM1) for 24 hours, the cell culture medium was replaced and subsequently treated with PDTC (100 μM) for a period of 24 hours prior to harvesting. Immunoblots were conducted on BV2 cells, including the vector, vector-PDTC, oeTREM1, and oeTREM1-PDTC groups, to assess TREM1, p-p65 to p65 ratio and p-IκBα to IκBα ratio (a-e). Immunoblots were also performed to assess the expression levels of NLRP3, cleaved-caspase-1, ASC, mature IL-1β, and mature IL-18 (f-k). Mean ± SEM values are presented. One-way ANOVA and Tukey's post hoc tests; ***p < 0.001 vs. the vector group and ^###^*p* < 0.001 vs. the oeTREM1 group

**Fig. 10:**
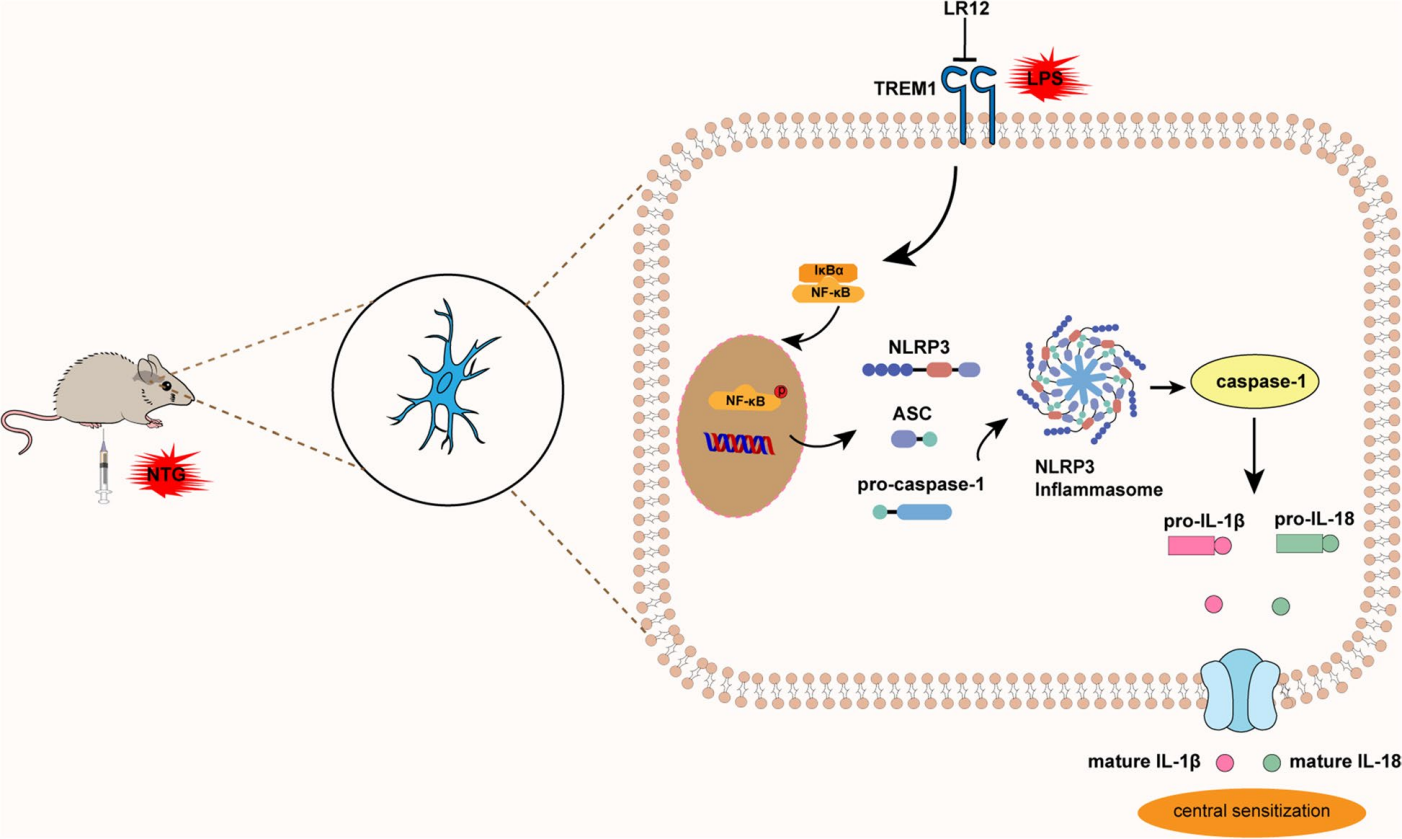
Schematic diagram of how microglia TREM1-mediated neuroinflammation contributes to central sensitization via the NF-κB pathway in a chronic migraine model

We successfully demonstrated that TREM1 exerts regulatory control over NLRP3 inflammasome activation via the NF-κB pathway. Subsequently, we aimed to ascertain whether TREM1 alone is sufficient for the regulation of NF-κB activity. We conducted an overexpression experiment in BV2 cells. Immunoblot analysis revealed that the transfection of TREM1 effectively augmented the expression level of TREM1 (Fig. 9a, b). TREM1 overexpression significantly upregulated the p-p65 to p65 and p-IκBα to IκBα ratios (Fig. 9c-e), indicating NF-κB signalling activation in TREM1-overexpressing BV2 cells. Moreover, PDTC treatment of TREM1-overexpressing BV2 cells effectively reduced NF-κB activation (Fig. 9c-e). Additionally, we observed that the expression levels of NLRP3 inflammasome components, mature IL-1β, and mature IL-18 were all elevated in the TREM1-overexpressing group, which could be effectively compensated by PDTC treatment (Fig. 9f-k). In summary, our findings demonstrate that TREM1 is both necessary and sufficient for activating NF-κB followed by subsequent NLRP3 inflammasome activation Fig. 10.

## Discussion

In our study, we identified TREM1 as a pivotal player in the pathophysiological processes of CM and elucidated its underlying mechanisms both in vitro and in vivo. Our findings confirmed that blocking TREM1 mitigates central sensitization, ameliorates pain symptoms associated with CM, suppresses the NF-κB pathway, and mitigates NTG-induced microglia and NLRP3 activation, as well as proinflammatory cytokines production. Collectively, our data highlight the crucial role of the TREM1-NF-κB axis as a key signalling mechanism regulating CM.

TREM1-mediated neuroinflammation has been shown to be involved in the pathophysiological processes of ischaemic stroke, subarachnoid haemorrhage, myocardial infarction, inflammatory bowel disease, and sepsis [30–34]. However, limited research has been conducted regarding its role in pain modulation. Suppression of TREM1 activity has been demonstrated to effectively attenuate inflammation and oxidative stress and alleviate pain hypersensitivity in models of spinal cord injury [16]. In our study, we observed a significant upregulation of TNC TREM1 expression in CM mice. Furthermore, we also observed a significant increase in TREM1 expression in BV2 microglia following LPS treatment. The TREM1 antagonist LR12 significantly alleviated mechanical and thermal hyperalgesia in CM mice. LR12 is a small molecule polypeptide that inhibits TREM1 activity [35]. Given our systematic drug administration approach, the potential involvement of other pain-associated regions, such as the trigeminal ganglia (TG), cannot be ignored. Currently, no TREM1 expression has been reported in the TG, and peripheral sensitization is closely related to CM [36, 37]. Therefore, our future work will focus on specifically targeting TREM1 within the TNC region. Our study revealed that TREM1 was localized in microglia but not neurons or astrocytes within the mouse TNC. Therefore, the LR12-induced changes in TREM1 in the TNC region originate from microglia. To our knowledge, this is the first investigation into both the expression and localization of TREM1 within the TNC. We speculate that the upregulation of microglia TREM1 contributes to CM development.

The pathophysiological mechanism underlying migraine is complex. In recent years, there has been growing recognition that the onset of migraine is strongly linked to central sensitization [38, 39]. CGRP, a neuropeptide and crucial regulatory factor in central sensitization, is widely recognized as a reliable biomarker for migraine [40]. After recurrent NTG administration, the expression of CGRP was significantly upregulated. Immunofluorescence staining revealed a shell-like appearance of CGRP, which was predominantly located in the superficial layer of TNC. Our findings are consistent with those of Zhou et al., who confirmed that CGRP is synthesized by the cell bodies of TG neurons and transported along nerve fibers to TNC, where it is involved in the processing of nociceptive information [27]. c-fos, a frequently used immediate early gene to indicate neuronal activation and closely linked with central sensitization [41], exhibits significantly increased expression in the TNC within our CM mice. The upregulation observed was significantly attenuated upon TREM1 blockade, providing compelling evidence for the involvement of TREM1 in the pathogenesis of central sensitization. Based on the neuronal production of c-fos and CGRP, we postulate that the inhibitory effect of TREM1 on these substances may arise from microglia-neuron interactions. Studies have indicated that microglia are capable of communicating with neurons via chemotaxis, where the branches of microglia extend towards nearby sensory neurons and trigger the production of various proinflammatory cytokines [10, 42]. Thus, the investigation of its potential molecular mechanism primarily centres on microglial activation and the inflammatory response.

Microglial activation can trigger the release of proinflammatory factors and enhance neuronal excitability, ultimately resulting in central sensitization [43, 44]. The pivotal role of TREM1 in amplifying inflammatory responses has been established in LPS-induced shock [45]. In ischaemic stroke and subarachnoid haemorrhage, inhibition of TREM-1 is accompanied by decreased microglia-associated neuroinflammation [30, 31]. In this study, we observed microglial activation in NTG-induced CM mice. The number of Iba1-labelled microglia and their fluorescence reactivity were significantly increased. Morphologically, the cell body of microglia exhibited enlargement, while their processes appeared shortened, which is consistent with previous findings [9, 10]. Blocking TREM1 ameliorated microglial activation and downregulated the expression of NLRP3 inflammasome components, leading to the attenuation of mature IL-1β and mature IL-18 levels. In vitro experiments showed that knockdown of TREM1 reduced the expression levels of inflammatory factors. Zhou et al. (2019) confirmed that NLRP3 is mainly expressed in microglia in the TNC region of migraine mice, and its expression is significantly upregulated after microglial activation, mediating the production of inflammatory factors and ultimately contributing to the development of migraine [23]. However, the underlying danger signal that triggers NLRP3 activation remains poorly understood. We discovered a predominant localization of NLRP3 in microglia within the TNC of migraine mice, which aligns with the findings reported by Jiying Zhou et al. Furthermore, we observed a robust co-localization between TREM1 and NLRP3 within the TNC of migraine mice. These findings suggest that NTG induces microglial activation via the TREM1 signalling pathway, leading to NLRP3 inflammasome activation and the subsequent production of various proinflammatory cytokines.

Studies have substantiated that NF-κB is involved in both inflammatory pain and neuropathic pain [46, 47], while valproate mitigates migraine symptoms in rats by inhibiting NF-κB activity [48]. We observed activation of NF-κB in CM mice, which was reversed by blocking TREM1. PDTC, a specific antagonist of NF-κB, significantly reduced pain hypersensitivity in CM mice and downregulated CGRP and c-fos expression. NF-κB inhibition has previously been demonstrated to facilitate the transition of microglia from an M1 to an M2 phenotype [49, 50]. Additionally, it has been reported that the NF-κB pathway contributes to NLRP3 transcription [51–53], suggesting its involvement in controlling microglial phenotype and NLRP3 inflammasome expression. We further investigated whether inhibiting NF-κB activity would impact microglial and NLRP3 inflammasome activation in the TNC region. The results were consistent with the LR12 group. In vitro studies have demonstrated that TREM1 overexpression activates the NF-κB pathway, resulting in NLRP3 activation, which could be reversed by PDTC treatment. These findings provide compelling evidence supporting the modulation of microglial and NLRP3 inflammasome activation by TREM1 through the NF-κB pathway. This regulatory mechanism ultimately influences the production of proinflammatory cytokines, thereby contributing to the pathogenesis of CM.

This experiment exhibits certain limitations. First, there are sex differences in migraine [54, 55], and oestrogen can affect the transmission of pain mediators [56]. We used male C57BL/6 mice to establish a model of migraine, and the effect of TREM1 in female animal models needs to be further studied. Second, the ligand of TREM-1 is poorly understood and may interact with other proteins. Third, the direct interaction between TREM1 and NF-κB has not been conclusively determined, and there may be other links between them. Fourth, we used systemic drug administration, and the changes in NF-κB in the TNC region may partially result from astrocytes and neurons, which may involve more complex intercellular crosstalk mechanisms. In the future, the development of a microglia-specific NF-κB promoter using gene editing technology may be a promising strategy for studying the role of microglia NF-κB in migraine. Further research is necessary to validate the precise mechanism of action of TREM1 in CM.

## Conclusion

In conclusion, we have elucidated the pivotal role of TREM1 in the pathogenesis of CM. Specifically, our data demonstrated that TREM1 exerts regulatory effects on microglial and NLRP3 inflammasome activation via modulation of the NF-κB signalling pathway. Moreover, TREM1 was implicated in CGRP and c-fos expression, thereby contributing to central sensitization (Fig. 10). These findings provide novel mechanistic insight into CM, suggesting that targeting TREM1 may be a promising therapeutic strategy for CM.

### Supplementary Information

**Additional file 1:** **Fig. S1.** Effects of varying doses of LR12 and PDTC on pain threshold in mice. (a-d) Basal and acute mechanical withdrawal thresholds of the hind paw measurement in the indicated groups. (n=5); values are the mean±SEM; two-way ANOVA and Tukey's post hoc tests. (a, b) *p< 0.05, **p < 0.01, ***^, ###, &&&^*p* < 0.001; 20, 50 and 100mg/kg LR12-treated groups, respectively, vs. the NTG group; ^^^*p* < 0.001 vs. the NTG-LR12 50mg/kg group. (c, d) **p < 0.01, ***^, ###,&&&^*p *< 0.001; 20, 40 and 100 mg/kg PDTC-treated groups, respectively, vs. the NTG group;^^*p* < 0.01, ^^^*p* < 0.001 vs. the NTG-PDTC 40mg/kg group.

**Additional file 2:** **Fig. S2. **NLRP3 exists mainly in microglia within the TNC of migraine mice and co-localizes well with TREM1. a Double immunofluorescence staining of NLRP3 and Iba1. b Double immunofluorescence staining of NLRP3 and TREM1. Scale bar: 20 μm.

**Additional file 3.** 

## Data Availability

All data used in this article are available from the corresponding author on reasonable request if necessary.

## Abbreviations

CM: Chronic migraine
NTG: Nitroglycerin
ASC: apoptosis-associated speck-like protein containing CARD
LR12: Nangibotide TFA
PDTC: Pyrrolidinedithiocarbamate ammonium
LPS: Lipopolysaccharides
IL-1β: Interleukin-1β
IL-18: Interleukin-18
ANOVA: Analysis of variance
CGRP: Calcitonin gene-related peptide
TNC: Trigeminal nucleus caudalis
HRP: Horseradish peroxidase
