# Supplementary figures and images for: Microglia TREM1-mediated neuroinflammation contributes to central sensitization via the NF-κB pathway in a chronic migraine model
Source: J Headache Pain. 2024 Jan 5;25(1):3. doi: 10.1186/s10194-023-01707-w (PMC10768449; doi:10.1186/s10194-023-01707-w)

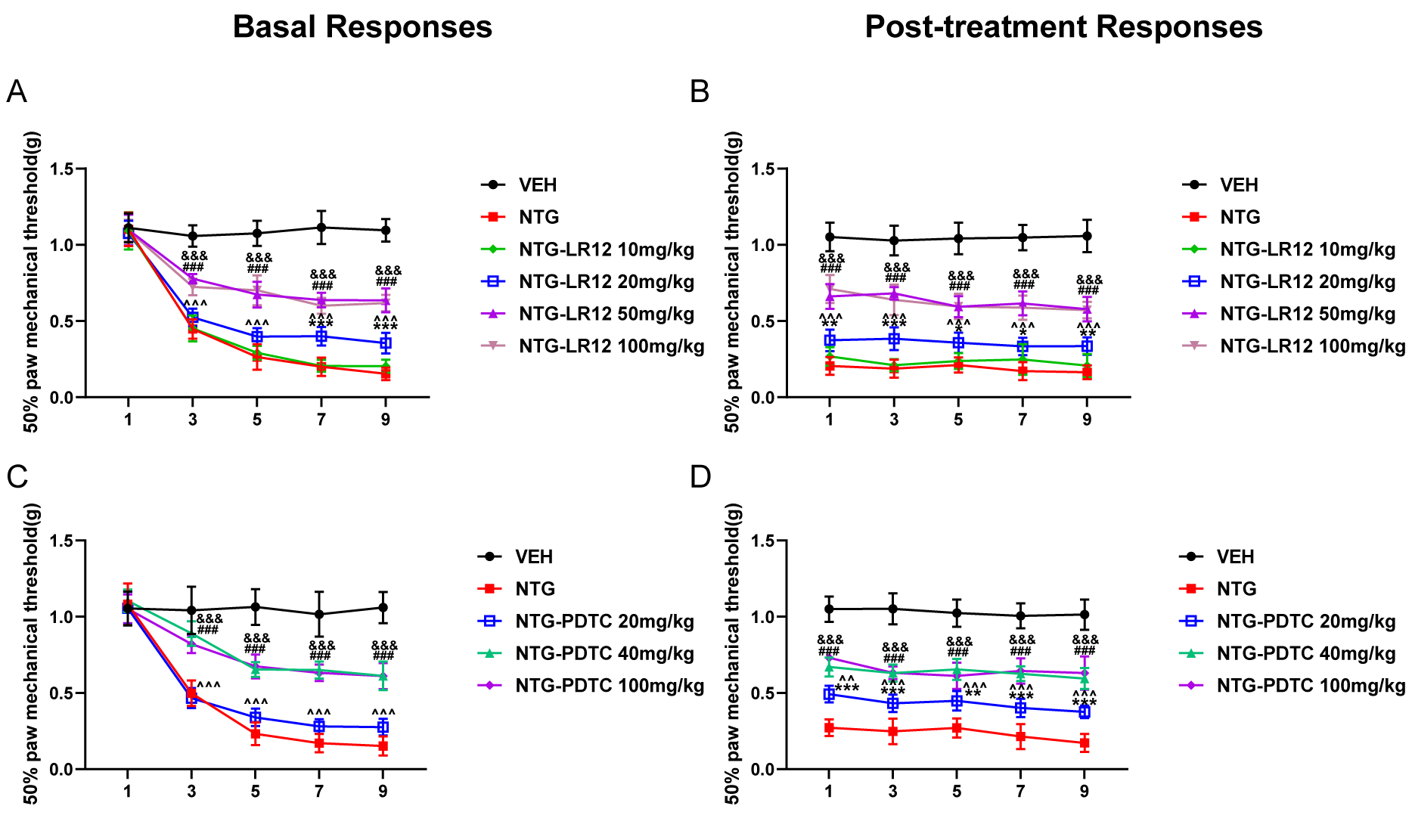

Supplement: Supplementary file 1 — Additional file 1: Fig. S1. Effects of varying doses of LR12 and PDTC on pain threshold in mice. (a-d) Basal and acute mechanical withdrawal thresholds of the hind paw measurement in the indicated groups. (n=5); values are the mean±SEM; two-way ANOVA and Tukey's post hoc tests. (a, b) *p< 0.05, **p < 0.01, ***, ###, &&&p < 0.001; 20, 50 and 100mg/kg LR12-treated groups, respectively, vs. the NTG group; ^^^p < 0.001 vs. the NTG-LR12 50mg/kg group. (c, d) **p < 0.01, ***, ###,&&&p < 0.001; 20, 40 and 100 mg/kg PDTC-treated groups, respectively, vs. the NTG group;^^p < 0.01, ^^^p < 0.001 vs. the NTG-PDTC 40mg/kg group. [file 10194_2023_1707_MOESM1_ESM.tif]

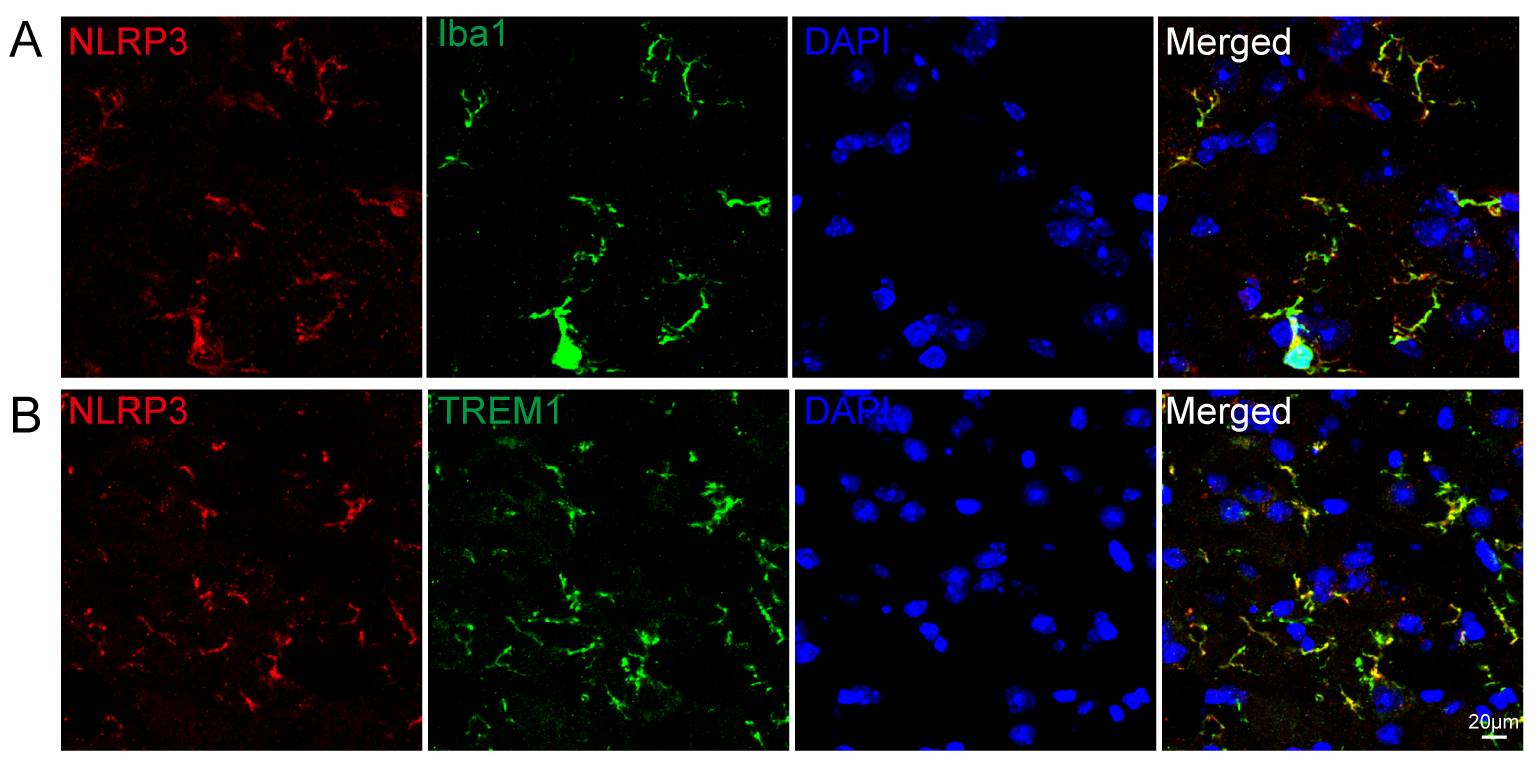

Supplement: Supplementary file 2 — Additional file 2: Fig. S2. NLRP3 exists mainly in microglia within the TNC of migraine mice and co-localizes well with TREM1. a Double immunofluorescence staining of NLRP3 and Iba1. b Double immunofluorescence staining of NLRP3 and TREM1. Scale bar: 20 μm. [file 10194_2023_1707_MOESM2_ESM.tif]

Fig. 1A

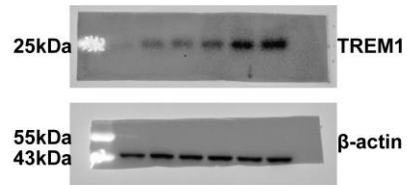

Fig. 3A

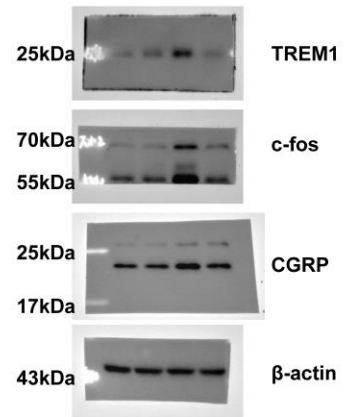

Fig. 4A

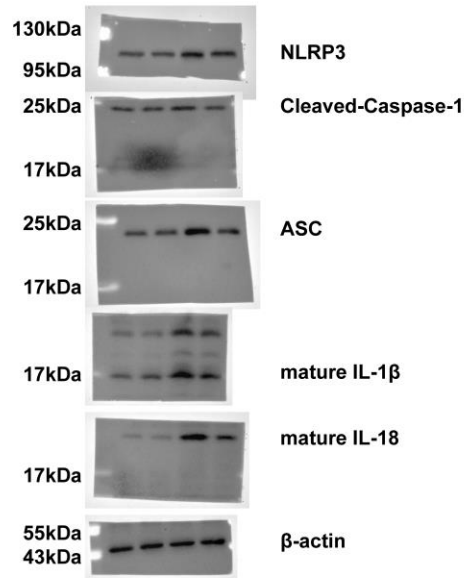

Fig. 5A

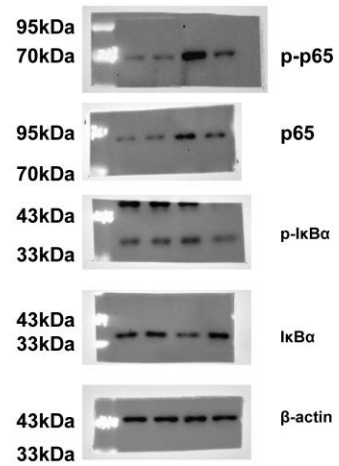

Fig. 5B

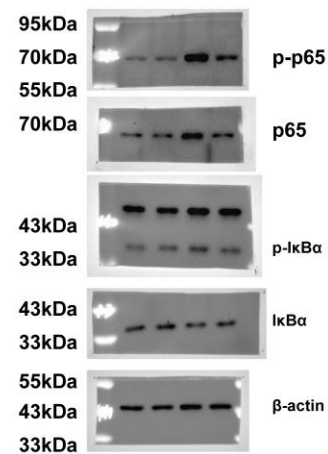

Fig. 5C

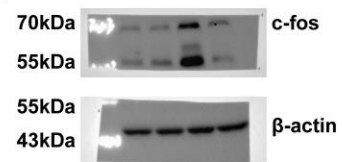

Fig. 5E

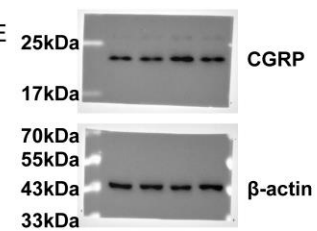

Fig. 7A

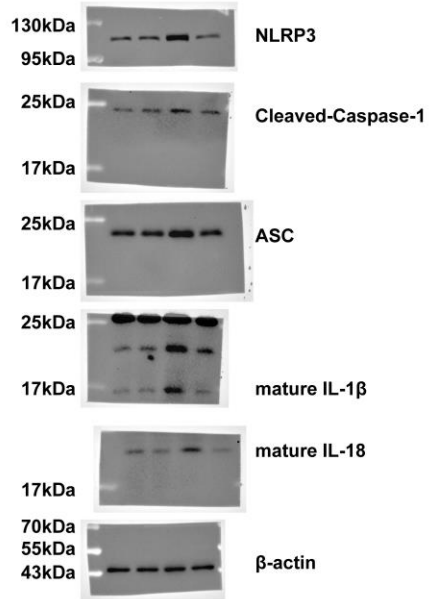

Fig. 8A

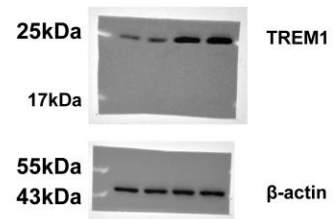

Fig. 8B

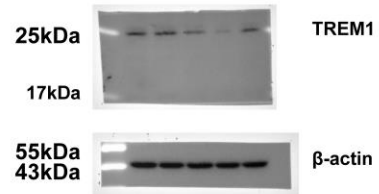

Fig. 8C

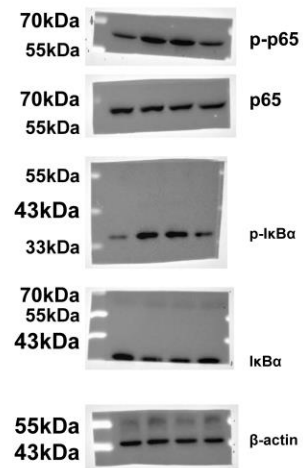

Fig. 8F

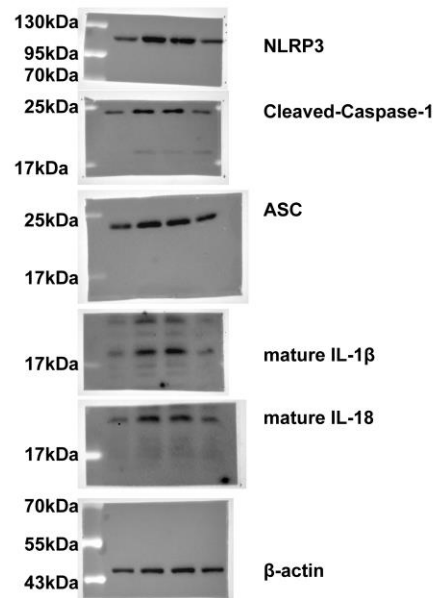

Fig. 9A

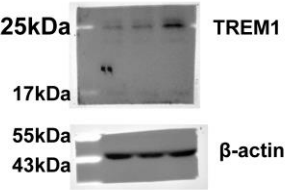

Figure. 9C

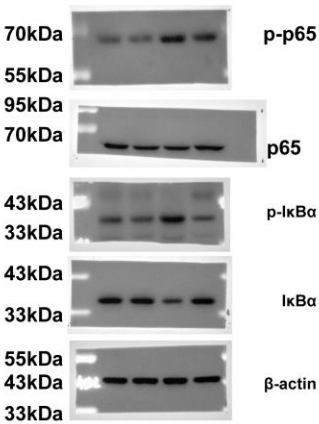

Fig. 9F

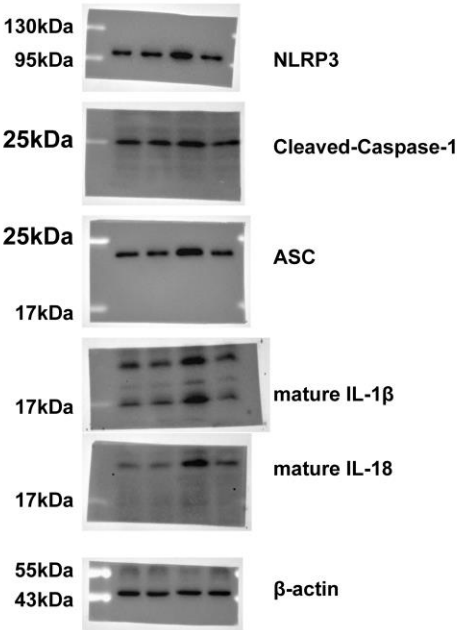

Supplement: Supplementary file 3 — Additional file 3. [file 10194_2023_1707_MOESM3_ESM.pdf]
